# Biomechanical Stimulation of Mesenchymal Stem Cells in 3D Peptide Nanofibers for Bone Differentiation

**DOI:** 10.3390/jfb17010052

**Published:** 2026-01-19

**Authors:** Faye Fouladgar, Robert Powell, Emily Carney, Andrea Escobar Martinez, Amir Jafari, Neda Habibi

**Affiliations:** Biomedical Engineering Department, University of North Texas, 3490 N Elm St., Denton, TX 76207, USA

**Keywords:** mesenchymal stem cells, dynamic stretching, bone tissue, osteogenic differentiation

## Abstract

Mechanical stimulation critically regulates mesenchymal stem cell (MSC) differentiation, yet its effects in three-dimensional (3D) environments remain poorly defined. Here, we developed a custom dynamic stretcher integrating poly(dimethylsiloxane) (PDMS) chambers to apply cyclic strain to human MSCs encapsulated in Fmoc-diphenylalanine (Fmoc-FF) peptide hydrogels—a fully synthetic, tunable extracellular matrix mimic. Finite element modeling verified uniform strain transmission across the hydrogel. Dynamic stretching at 0.5 Hz and 10% strain induced pronounced cytoskeletal alignment, enhanced actin stress fiber formation (coherency index ≈ 0.85), and significantly increased proliferation compared to static or high-frequency (2.5 Hz, 1%) conditions (coherency index ≈ 0.6). Quantitative image analysis confirmed strain-dependent increases in coherency index and F-actin intensity, indicating enhanced mechanotransductive remodeling. Biochemical assays and qRT–PCR revealed 2–3-fold upregulation of osteogenic markers—*RUNX2*, *ALP*, *COL1A1*, *OSX*, *BMP*, *ON*, and *IBSP*—under optimal strain. These results demonstrate that low-frequency, high-strain mechanical loading in 3D peptide hydrogels activates RhoA/ROCK and YAP/TAZ pathways, driving osteogenic differentiation. The integrated experimental–computational approach provides a robust platform for studying mechanobiological regulation and advancing mechanically tunable biomaterials for bone tissue engineering.

## 1. Introduction

Bone graft procedures are increasingly employed in traumatology, tumor surgery, spinal surgery, infection management, and revision arthroplasty [1,2,3]. Human mesenchymal stem cells (hMSCs) are multipotent progenitors with the capability to differentiate into osteogenic, chondrogenic, myogenic, and adipogenic lineages [4,5,6]. MSCs hold significant therapeutic potential in patients with fractures, as they can enhance bone regeneration, accelerate healing, and improve outcomes in cases of delayed union or nonunion [7]. MSCs contribute to bone repair through their ability to differentiate into osteoblasts, secrete pro-regenerative growth factors, and modulate the local immune environment, making them a promising adjunct to conventional bone grafting techniques [8,9]. The extracellular microenvironment plays a central role in directing hMSC fate, with both biochemical and biophysical cues contributing to lineage specification [10,11]. Among these cues, mechanical stimulation has emerged as a critical regulator of osteogenic differentiation.

Previous studies show that hMSCs can sense substrate deformation through integrin-mediated adhesions that relate the extracellular matrix (ECM) to the cytoskeleton [12,13]. Mechanical stretching activates mechanotransduction pathways such as focal adhesion kinase (FAK), ERK, and p38 MAPK, leading to the upregulation of osteogenic markers including alkaline phosphatase (ALP), osteocalcin (OCN), and Runx2 [14,15,16]. Studies have reported enhanced osteogenic differentiation when hMSCs are subjected to cyclic stretching in two-dimensional (2D) culture systems [10,13,17]. However, 2D platforms do not adequately mimic the three-dimensional (3D) microenvironment of bone tissue, which provides cells with spatial, mechanical, and biochemical signals that are essential for physiological function [18,19,20]. Although the effects of mechanical stimulation and 3D culture have been studied independently [21,22,23], the combination of dynamic stretching with 3D hydrogels remains largely unexplored. This gap is significant, as the integration of biomechanical cues with a tunable synthetic ECM mimic may more closely replicate the conditions that drive osteogenesis in vivo [24,25,26,27].

Natural ECM proteins such as collagen, fibronectin, and laminin have been widely used to support hMSC adhesion and osteogenesis on a 2D substrate [28,29,30]. Yet, these biomaterials suffer from limitations including batch-to-batch variability, poor tunability, and susceptibility to enzymatic degradation. In contrast, self-assembling peptide hydrogels offer a chemically defined and modular system with controllable stiffness, nanofibrous architecture, and reproducibility [31,32,33]. Prior studies demonstrate that certain peptide amphiphiles (PAs) can self-assemble into aligned nanofiber gels capable of directing the orientation of mesenchymal stem cells (MSCs). These aligned PA noodles were further used to encapsulate and mechanically influence MSCs in 3D culture, enabling their alignment and stretch [34]. Among assembling peptides, Fmoc-diphenylalanine (Fmoc-FF) hydrogels provide unique advantages, including high biocompatibility and the ability to promote cellular alignment, a feature particularly relevant for organized tissue formation [35,36]. Previously, we demonstrated that encapsulating hMSCs in peptide hydrogels enhances cell viability and promotes MSC spreading and elongation [31,36]. In this study, we examined the response and osteogenicity of hMSCs encapsulated in Fmoc-FF peptide hydrogels subjected to dynamic cyclic stretching. This approach provides new insight into the role of mechanotransduction in 3D environments and offers a versatile platform for osteogenesis tissue engineering.

## 2. Experimental Section

### 2.1. Materials

Bone-marrow-derived mesenchymal stem cells (MSCs) were purchased from American Type Culture Collection (ATCC) (USA) and maintained in Dulbecco’s Modified Eagle Medium (DMEM; Grand Island Biological Company (Gibco, Grand Island, NY, USA), Cat. No. 11-965-092) supplemented with 9% fetal bovine serum (FBS; Gibco, Cat. No. 26140079) and 1% penicillin–streptomycin (Gibco, Cat. No. 15140122). Trypsin-EDTA and Fmoc-Phenylalanyl- phenylalanine (Phe-Phe catalog no J60043.03) were also obtained from Thermo Fisher Scientific (Waltham, MA, USA). Hexafluoroisopropanol (HFP/ 1,1,1,3,3,3-Hexafluoro-2-propano catalog no 105,228) was obtained from Millipore Sigma (Burlington, MA, USA). Alexa Fluor 488 Phalloidin (catalog no A12379), DAPI (catalog no D1306), Alizarin Red assay kit (catalog no A12379), alkaline phosphatase assay kit (catalog no 637,944), osteocalcin immunostaining (catalog no OSTCLN-FITC), ascorbate-2 phosphate (Thermo Fisher Scientific, catalog number A25215 G), 10 M dexamethasone (A17590.14, Thermo Fisher Scientific), *β*-glycerophosphate (J62121.AD Thermo Fisher Scientific), MTT (catalog no V13154), and LIVE-Dead (catalog no L32250) were also obtained. Sylgard Silicone Elastomer Catalog No. 50-822-180 was purchased from Fisher Scientific and was used to produce PDMS chambers. Mightyzap Micro/Mini Linear Motor Actuators for the development of the stretcher were purchased from Robotshop, with specifications of w/22 mm Stroke, 35 N Force, 28 mm/S Speed, and 12 V Voltage. Fluorescence imaging and quantitative analyses were conducted using an Agilent BioTek (Winooski, VT, USA) Synergy LX Multimode Reader, an Olympus^®^ microscope with CellSens^®^ V3.2 software, a Keyence BZ-X810 fluorescence microscope, and ImageJ 1.54p. qPCR was performed using Thermo Fisher Applied Biosystems (Foster City, CA, USA) QuantStudio 3 Real-Time PCR System, in 96-well format.

### 2.2. Design of Dynamic Cell Stretcher with PDMS Cell Chamber

**Figure 1:**
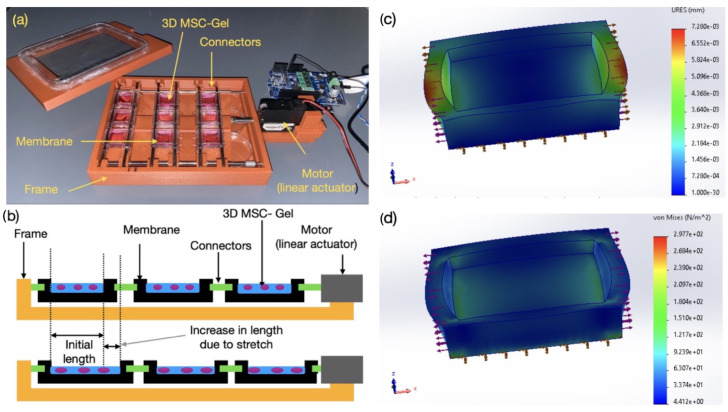
Schematic and configuration of the customized stretching platform for 3D MSC culture. (**a**) Photograph of the assembled apparatus with integrated PDMS chambers positioned within a 3D-printed frame and connected to a linear actuator for cyclic mechanical loading. MSCs encapsulated within Fmoc-FF hydrogels were housed inside the chambers. (**b**) Cross-sectional representation of the stretcher showing how the PDMS membrane is anchored to connectors attached to both the frame and the motor. Linear motion applies uniaxial deformation to the hydrogel constructs. (**c**) Finite element simulation illustrating total displacement (URES) across the PDMS membrane during stretching. (**d**) Associated von Mises stress map highlighting regions of stress concentration at the membrane–connector interfaces.

Commercially available cell stretcher systems provide limited chamber capacity, which restricts the number of experimental replicates. To overcome this limitation, a custom mechanical stretcher incorporating polydimethylsiloxane (PDMS) culture chambers was designed and fabricated. The structural frame holding the PDMS rods was printed using a Bambu Lab (Shenzhen, China) X1E 3D printer. Each platform contains three unhinged rods and three stationary rods that are fixed in place and unable to be moved. Positioned between each pair of unhinged (connectors) and stationary rods are three parallel PDMS chambers, giving a total of nine chambers per platform. All unhinged rods are mechanically linked to a linear motor shaft (from mightyZAP Linear Motor); therefore, movement of the motor shaft drives the motion of the unhinged rods, while the stationary rods remain fixed. This differential motion stretches the PDMS chambers. The motor is controlled by an Arduino hmicrocontroller, which regulates both the frequency and stroke length of the motor shaft (Figure 1a,b).

To fabricate the PDMS chambers, the PDMS Elastomer Kit (Fisher Scientific (Waltham, MA, USA), Cat. No. NC9285739) was used. After mixing components A and B, the mixture was stirred thoroughly and centrifuged at 1200 rpm for 10 min to remove trapped air. The PDMS prepolymer, which retained suitable viscosity, could be stored at 4 °C for as long as one month. For molding, the mixture was poured into a 3D-printed mold designed with openings for metal rods that prevented overflow. After transferring the PDMS solution to the mold, the mixture was degassed under vacuum and then cured overnight at 75 °C. Completed chambers were affixed to the stretching platform (Figure 1) prior to cell loading.

### 2.3. Dynamic Stretching of MSC in 3D Peptide Hydrogels

Human MSCs isolated from bone marrow (Fisher Scientific, Cat. No. SCC034) were cultured between passages 3 and 6 in α-MEM supplemented with 1% L-glutamine, 16% FBS, and 1% antibiotic solution. Cells at confluence were trypsinized with Trypsin–EDTA and resuspended in fresh medium. For three-dimensional encapsulation, cells were incorporated into Fmoc-FF hydrogels. In each microplate well, 100 μL of peptide solution was deposited, followed by addition of cell-containing medium. Gentle pipetting ensured uniform hydrogel formation. The cell/gel was transferred into the PDMS stretching chambers, and medium was replenished every two days using cell-insert. Control gels were prepared identically but maintained without mechanical loading.

Cultures were kept in growth medium for the first three days before switching to osteogenic induction medium (OIM), consisting of α-MEM-LG supplemented with 10% FBS, dexamethasone, ascorbate-2-phosphate, and *β*-glycerophosphate (all from Thermo Fisher). Mechanical stretching was administered for 14 days at 0.5–2 Hz, 6 h per day, with a uniaxial strain amplitude of 0.1%. Cell viability was evaluated using the MTT assay, where cells were incubated with MTT reagent to allow viable cells to reduce it to insoluble formazan crystals. The crystals were dissolved in solvent, and absorbance was measured at 570 nm using a microplate reader to quantify cell viability.

### 2.4. Assessing Osteogenic Differentiation

Mineralization was determined using Alizarin Red assay. The culture medium was aspirated, and samples were washed with PBS and fixed in 4% paraformaldehyde. Constructs were then incubated with Alizarin Red, which binds calcium-rich deposits. Excess dye was extracted using acetic acid to solubilize the bound complex, and the resulting solution was collected. Absorbance at 590 nm was measured to quantify calcium accumulation and infer osteogenic progression.

Early differentiation was evaluated through alkaline phosphatase (ALP) staining on day 14. Following medium removal, samples were fixed and rinsed with PBS. An ALP staining reagent, prepared following the manufacturer’s directions, was added, and samples were kept in the dark during incubation. Positively stained colonies appeared red, whereas undifferentiated cells remained unstained. Colony counts were used to assess ALP activity.

### 2.5. Immunofluorescence Staining

F-actin organization was examined through immunofluorescence. At day 14 of the stretching regimen, cells were fixed in 4% paraformaldehyde for 20 min, washed twice with PBS containing 0.05% Tween-20, and permeabilized for 5 min using 0.1% Triton X-100. Samples were treated in 1% BSA for 30 min and incubated with Alexa Fluor 488 Phalloidin (1:1000) for 1 h to label filamentous actin. DAPI was used to label cell nuclei, and imaging was performed with an Olympus IX73 fluorescence microscope (Olympus, Tokyo, Japan). Cell viability was assessed using a Live/Dead assay, in which living cells were stained with calcein-AM (green fluorescence) and dead cells with ethidium homodimer-1 (red fluorescence) according to the manufacturer’s protocol. After incubation, samples were imaged using fluorescence microscopy, and live/dead cell ratios were quantified using image analysis software.

Osteocalcin expression was visualized by immunostaining. Constructs underwent fixation with 400 μL of 4% paraformaldehyde (pH 7.4) at 37 °C for 10 min, followed by three PBS washes and a 10–15 min permeabilization in 0.1% Triton X-100/PBS. An FITC-conjugated osteocalcin antibody (1:250) was applied, and samples were incubated for 1–2 h at room temperature or overnight at 4 °C in darkness. Images were obtained using a microscope equipped with a green fluorescence filter set (excitation 490 nm, emission 520 nm).

### 2.6. RNA Extraction and Real-Time Quantitative RT-qPCR

MSC constructs cultured within hydrogels under static or stretched conditions were processed for RNA isolation using TRIzol^TM^ Reagent (Thermo Fisher Scientific). After medium removal, 1 mL of TRIzol was added directly to each well. Cells were scraped, transferred to 1.5 mL tubes, and allowed to lyse for 2–3 min. Chloroform (200 μL) was added, and samples were vortexed, incubated briefly, and centrifuged. The aqueous phase was recovered, combined with an equal volume of isopropanol, and centrifuged for 30 min at 12,000×g to precipitate RNA. Pellets were washed with 75% ethanol, air-dried, and dissolved in 30–50 μL TE buffer before storage at −80 °C.

RNA yield and purity were evaluated using a Synergy LX reader and Take3 microvolume plate. Samples with A_260/280_ ≥ 1.7 were treated with ezDNase and reverse-transcribed using the SuperScript^TM^ IV VILO^TM^ kit (Thermo Fisher Scientific). Reverse transcription was carried out on a MiniAmp Thermal Cycler with the following steps: 25 °C (10 min), 50 °C (10 min), and 85 °C (5 min). Primers were designed using NCBI Primer-BLAST (https://www.ncbi.nlm.nih.gov/tools/primer-blast/, accessed on 18 November 2025) to span exon junctions, produce 70–200 bp amplicons, and possess melting temperatures near 62 °C with 40–60% GC content. Oligonucleotides were resuspended to 80,000 nM and diluted to 8000 nM for qPCR. qPCR reactions were performed using PowerTrack SYBR Green Master Mix (Thermo Fisher Scientific) on a QuantStudio 3 Real-Time PCR System. Each 10 μL reaction contained 5 μL SYBR mix, 1 μL cDNA, 0.85 μL each of forward and reverse primers, and nuclease-free water. Plates were sealed with optical film, centrifuged briefly, and run using the recommended SYBR cycling program. Gene expression was quantified using the ΔΔCt method, with GAPDH as the internal control. Relative expression levels were compared among the various peptide-treated and stretched samples.

Primer efficiency for quantitative real-time PCR (qPCR) was optimized prior to gene expression analysis for all target genes, including *COL1A1*, *BMP2*, *ON (osteonectin)*, *RUNX2*, *ALP*, *OSX*, and *IBSP*. For each gene, several primer combinations were designed and tested, comprising three forward and three reverse primers. Candidate primer pairs were screened using serial dilutions of cDNA to evaluate amplification efficiency, linearity, and specificity. Primer pairs demonstrating acceptable amplification efficiency and minimal Ct variability were selected for subsequent experiments, while those showing non-specific amplification or primer–dimer formation were excluded. The final primer pairs used in this study are listed in Table 1.

Amplification specificity was verified by melt curve analysis performed at the end of each qPCR run. All selected primer pairs produced a single, well-defined melt peak, indicating specific amplification of the target amplicons, with no evidence of primer–dimer formation. No amplification was detected in no-template controls. Relative gene expression levels were calculated using the ΔΔCt method, normalized to the housekeeping gene, and expressed as fold change relative to control samples.

### 2.7. Statistical Analysis

Data are presented as mean ± SD (n replicates = 3). Statistical analyses were performed using one-way ANOVA for multiple-group comparisons and Student’s *t*-test for pairwise comparisons. Statistical significance was denoted as follows: ns, not significant; *, p<0.05; **, p<0.01; ***, p<0.001.

## 3. Results and Discussion

### 3.1. Finite Element Modeling (FEM) of 3D Hydrogel Stretching in PDMS

Finite element modeling (FEM) was conducted to evaluate the deformation and stress distribution within the PDMS membrane during cyclic uniaxial stretching (Figure 1c,d). The simulation geometry replicated the actual dimensions of the chamber and included boundary constraints at the frame and connector regions. A uniform displacement corresponding to the actuator-induced strain was applied along one edge, while the opposite side was fixed to mimic experimental conditions. The total displacement contour (Figure 1c) revealed a non-uniform strain distribution, with the highest deformation localized near the connector attachments, indicating effective strain transmission to the central region where the hydrogel constructs reside. The applied strain regime falls within physiologically relevant tensile ranges reported for musculoskeletal tissues, where low strains (0.1–1% or 0.001 mm) are associated with osteogenic signaling, while moderate cyclic strains (2–6%) regulate tendon- and ligament-like matrix organization. Through maintaining strain amplitudes within this window and minimizing stress concentrations, the platform enables controlled activation of MSC mechanotransduction pathways without inducing non-physiological or damaging mechanical cues. Figure 1d presents the von Mises stress distribution, highlighting localized stress concentrations at the membrane–connector interface with progressive dissipation toward the central zone. These results confirm that the mechanical loading is predominantly uniaxial, with minimal out-of-plane deformation, ensuring controlled delivery of cyclic tensile strain to the encapsulated 3D MSC–Gel constructs. The FEM outcomes were consistent with the observed experimental performance of the stretcher, validating its design for reproducible mechanobiological stimulation studies.

The von Mises stress distribution indicates that stresses within the central hydrogel culture region remain within physiologically relevant ranges for soft tissue-embedded MSCs (typically on the order of 0.1–5 kPa), while higher stresses are confined to the membrane–connector interface. This stress regime is consistent with native extracellular matrix stresses experienced by MSCs in musculoskeletal tissues and is well below reported thresholds associated with stress-induced apoptosis or loss of viability (generally >10–20 kPa). The gradual dissipation of stress toward the cell-laden region ensures uniform mechanical stimulation, thereby supporting sustained MSC mechanotransduction and preserving cellular integrity during cyclic tensile loading.

It is important to note that the region of interest for all biological experiments corresponds to the central portion of the PDMS membrane modeled in the FEM, which is fully occupied by the cell-laden Fmoc-FF hydrogel. As shown by the displacement and stress contours, this central region experiences a nearly uniform uniaxial strain with minimal transverse gradients. Therefore, the simulated strain field directly represents the mechanical environment sensed by the encapsulated MSCs. The analysis assumed homogeneous, isotropic PDMS, which is appropriate for capturing the overall mechanical behavior, although minor local variations arising from curing or bonding may lead to small deviations in strain transfer. In addition, the thickness of the 3D peptide hydrogel may influence nutrient and oxygen transport over extended culture periods. While FEM confirmed uniform in-plane strain, mechanical transmission through the full thickness of the hydrogel may vary slightly.

### 3.2. MSC Proliferation and Reorientation Stretched in Hydrogels

**Figure 2:**
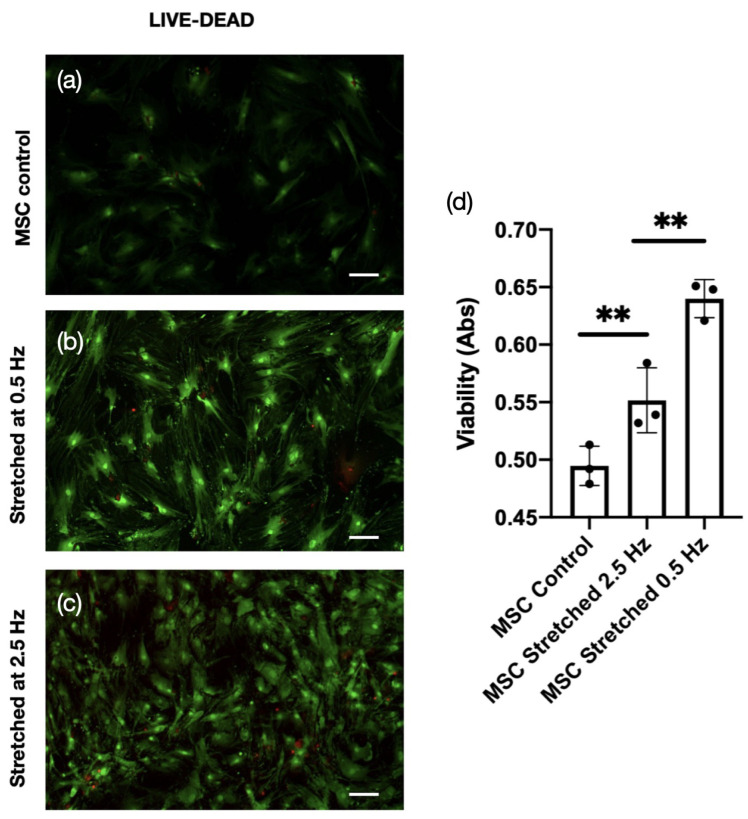
Live/dead cell viability analysis of mesenchymal stem cells (MSCs) encapsulated in Fmoc-FF peptide hydrogels under static and dynamic stretching conditions. Fluorescence images show live cells stained green (calcein AM) and dead cells stained red (ethidium homodimer-1) for (**a**) static MSC control, (**b**) MSCs stretched at 0.5 Hz, (**c**) MSCs stretched at 2.5 Hz, and (**d**) viability of cells measured by MTT assay. Corresponding quantitative analyses (right panels) indicate a significant increase in cell viability under dynamic stretching compared to static controls, with higher viability observed at both 0.5 Hz and 2.5 Hz frequencies. Scale, 200 μm. **, p<0.01.

We previously demonstrated that culturing hMSCs within peptide hydrogels under static conditions enhances cell growth compared to conventional 2D culture. In the present study, we assessed cell viability under dynamic mechanical stimulation in 3D hydrogels using MTT assays at two different strain regimens: 0.5 Hz with 10% strain and 2.5 Hz with 1% strain (Figure 2).

The selected strain amplitudes and frequencies were based on physiologic ranges reported for bone and soft tissue loading. A 10% strain amplitude approximates cyclic deformation experienced during daily musculoskeletal activity, whereas 1% strain represents a subthreshold mechanical input. Likewise, 0.5 Hz reflects slow, physiologic movement, while 2.5 Hz represents high-frequency loading that often results in reduced mechanosensitive signaling. These parameters enabled direct evaluation of how frequency and magnitude interact to regulate MSC mechanotransduction. Both stretching conditions resulted in significantly higher viability compared to non-stretched controls, suggesting that cyclic strain promotes activation of growth and proliferative pathways. When the two frequencies were compared, cells subjected to 0.5 Hz exhibited greater viability, suggesting that lower-frequency, higher-strain loading provides a more supportive mechanical environment. The influence of uniaxial strain on F-actin filament organization was assessed under conditions of 2 weeks of stimulation, with a frequency of (0.5 Hz, 10% strain). To assess how cytoskeletal structures contribute to MSC remodeling under cyclic stretch, F-actin fiber images were captured both before and after mechanical loading. Confocal microscopy revealed that actin stress fibers reoriented predominantly perpendicular to the direction of applied strain, indicating a cellular adaptation mechanism aimed at reducing mechanical stress on the cell body. The precise molecular mechanisms governing MSC proliferation in response to mechanical stretch remain poorly understood, requiring further in-depth investigation. Nonetheless, our findings, together with reports from other studies, indicate that mechanical stimulation enhances the proliferative capacity of MSCs. This proliferative response appears to be coincided by cytoskeletal reorganization and alignment of structural components within the cell structure, which may serve as a precursor to lineage-specific differentiation. MSCs’ viability is influenced by the frequency of applied mechanical stretch. Moderate frequencies (e.g., 0.5–2 Hz) are often associated with improved cell survival and balanced mechanotransduction, whereas excessively high frequencies (>5 Hz) may impose mechanical stress that disrupts cytoskeletal integrity and reduces cell viability. Conversely, very low frequencies may provide insufficient mechanical cues to elicit a significant biological response. Overall, while stretching itself promotes MSC viability compared to static conditions, frequency within a physiological range seems to fine-tune rather than determine the viability outcomes of MSCs.

### 3.3. Quantitative Analysis of Cytoskeletal and Nuclear Responses Under Cyclic Stretch

**Figure 3:**
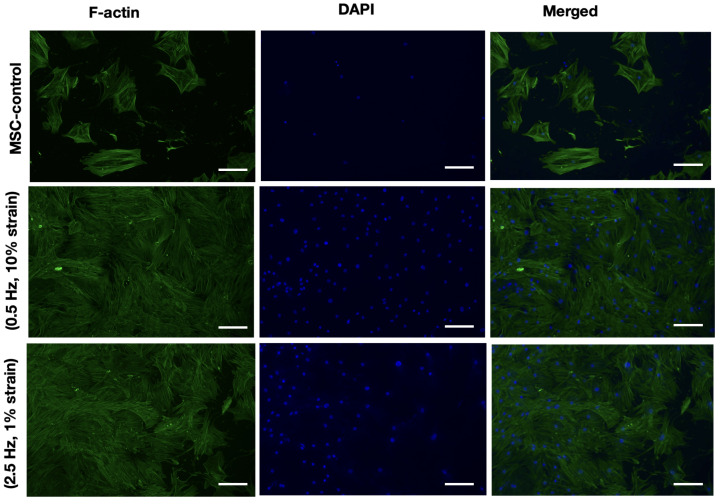
Fluorescence images showing F-actin (green) and nuclei (DAPI, blue) staining of MSCs encapsulated in Fmoc-FF peptide hydrogels under static and dynamic stretching. Cells subjected to 0.5 Hz, 10% strain displayed enhanced actin fiber alignment and elongation compared to control and 2.5 Hz, 1% strain conditions, indicating strain-dependent cytoskeletal organization and mechanosensitive remodeling. Scale, 100 μm.

To measure how cyclic mechanical loading influences MSCs, fluorescence micrographs of F-actin and DAPI-labeled cells under different strain conditions were analyzed. The three experimental groups consisted of unstimulated MSC controls, cells exposed to 10% strain at 0.5 Hz, and cells subjected to 1% strain at 2.5 Hz. Image analysis was performed in Fiji/ImageJ to assess three primary mechanobiological parameters: cytoskeletal alignment, F-actin intensity, and cell density (Figure 3).

Cytoskeletal orientation was quantified using the Directionality and OrientationJ plugins in Fiji [37]. Using this software, fluorescence images of F-actin were converted to grayscale and analyzed to detect linear features corresponding to actin fibers. The Directionality plugin computes the local orientation of these structures using a Fourier transform-based method and generates a histogram of fiber angles relative to a reference axis, providing a measure of overall alignment. OrientationJ performs structure tensor analysis to calculate pixel-wise orientation and coherence, allowing quantification of both the preferred fiber direction and the degree of cytoskeletal anisotropy. These analyses enable objective assessment of actin alignment and reorganization in response to applied mechanical strain (Table 2).

Following background subtraction and thresholding, the distribution of filament orientations was extracted and expressed as an angular histogram. From these data, a coherency index (*CI*) was computed to describe the degree of cytoskeletal alignment. The coherency index is defined as

(1) $$CI=1-\frac{\mathrm{Circular}\;\mathrm{Variance}}{180^{{}^{\circ}}},$$

where the circular variance represents the spread of orientation angles relative to the mean direction. A *CI* value of 1 indicates perfect alignment, while 0 represents a random orientation distribution. The results revealed that unstrained MSCs exhibited a low degree of organization (*CI* ≈ 0.25), consistent with the random orientation of actin filaments in the control group. In contrast, cells exposed to 0.5 Hz at 10% strain demonstrated a pronounced cytoskeletal reorganization, yielding a coherency index of approximately 0.85. Cells exposed to 2.5 Hz at 1% strain exhibited intermediate alignment (*CI* ≈ 0.6), indicating that strain amplitude played a more dominant role than frequency in cytoskeletal remodeling.

F-actin intensity was quantified as a proxy for intracellular tension and stress fiber formation. Mean fluorescence intensity values were obtained from the F-actin channel after consistent thresholding across samples. For comparative analysis, each intensity value was normalized to the mean of the control group according to

(2) $$I_{\mathrm{norm}}=\frac{I_{\mathrm{condition}}}{I_{\mathrm{control}}},$$

where $I_{\mathrm{condition}}$ and $I_{\mathrm{control}}$ denote the mean fluorescence intensity of the experimental and control samples, respectively. The normalized intensity increased from 1.0 in the control group to 1.5 under 0.5 Hz, 10% strain, and to 1.2 under 2.5 Hz, 1% strain. The enhancement of F-actin fluorescence under cyclic strain reflects an increase in stress fiber polymerization, suggesting that mechanical loading promotes cytoskeletal reinforcement and actomyosin contractility.

The number of DAPI-labeled nuclei per unit area was quantified to evaluate cell density. The blue channel images were thresholded, and nuclei were segmented and counted using the *Analyze Particles* function with a size filter range of 50–500 μm^2^ to exclude debris and overlapping nuclei. The resulting values were normalized to the control, yielding a relative cell density of approximately 1.0 for the control, 1.5 for the 0.5 Hz, 10% strain condition, and 1.3 for the 2.5 Hz, 1% strain condition. The increase in cell number suggests enhanced adhesion or proliferation under dynamic mechanical stimulation, consistent with previous findings that moderate cyclic strain can activate mechanotransductive pathways linked to proliferation and differentiation.

The combined quantitative results indicate that cyclic mechanical loading elicits frequency- and strain-dependent reorganization of the MSC cytoskeleton. Low-frequency, high-amplitude strain (0.5 Hz, 10%) induced the most pronounced actin alignment and stress fiber development, while high-frequency, low-amplitude strain (2.5 Hz, 1%) produced moderate alignment and cytoskeletal tension. These trends support a model in which the magnitude of mechanical deformation acts as the primary regulator of actin remodeling, while frequency modulates the dynamic adaptation response. Changes in F-actin intensity under cyclic strain provide important mechanistic insight into how MSCs activate mechanotransductive pathways during mechanical loading. At the mechanistic level, the observed cytoskeletal and nuclear responses can be interpreted within the framework of mechanotransduction pathways that regulate MSC behavior. Cyclic deformation of the actin cytoskeleton generates tension that activates RhoA and its downstream effector ROCK, promoting actomyosin contractility and stress fiber assembly. The pronounced alignment and thickening of F-actin bundles under 10% strain at 0.5 Hz are therefore indicative of elevated RhoA/ROCK activity, which enhances cytoskeletal tension and focal adhesion maturation. These mechanical cues are relayed to the nucleus through the LINC (linker of nucleoskeleton and cytoskeleton) complex, which connects the contractile actin cytoskeleton to the nuclear envelope. As a consequence, the nuclear envelope experiences tensile forces that influence chromatin organization and transcriptional activity.

One of the principal downstream effectors of this cytoskeletal–nuclear coupling is the YAP/TAZ signaling axis. Under increased mechanical loading, nuclear deformation facilitates the translocation of YAP and TAZ transcriptional coactivators into the nucleus, where they promote the expression of genes associated with proliferation and osteogenic differentiation. The elevated nuclear density observed under cyclic strain, particularly at 0.5 Hz and 10% strain, aligns with this mechanistic interpretation, suggesting that mechanical stimulation enhanced proliferation through YAP/TAZ-mediated mechanosignaling. In contrast, cells subjected to 2.5 Hz and 1% strain exhibited more moderate alignment and actin intensity, implying that high-frequency but low-amplitude stimulation elicited subthreshold activation of these pathways. Overall, the finding demonstrates that the observed increase in F-actin alignment, particularly under 0.5 Hz and 10% strain, reflects enhanced stress fiber polymerization and actomyosin contractility—hallmarks of RhoA/ROCK pathway activation. Mechanical deformation of the hydrogel induces tension across integrin–ECM adhesions, which stimulates RhoA activation and promotes the assembly of thicker, more aligned actin bundles. This cytoskeletal reinforcement increases intracellular tension and facilitates force transmission to the nucleus through the LINC complex, thereby modulating nuclear shape and chromatin organization. As tension rises, YAP/TAZ mechanosensors translocate into the nucleus, where they enhance transcription of proliferation- and osteogenesis-related genes. Thus, the elevated F-actin intensity measured in stretched samples is not merely a structural change but a quantitative indicator of heightened mechanotransductive signaling that primes MSCs for osteogenic differentiation.

The quantitative image analysis, combined with the mechanotransductive interpretation, underscores the sensitivity of MSCs to both strain magnitude and frequency. High-magnitude, low-frequency stretching effectively engages cytoskeletal tension and nuclear mechanosignaling, driving an adaptive cellular response characterized by actin alignment, enhanced proliferation, and likely lineage priming toward a mechanically robust phenotype. This multiscale coupling between external strain and intracellular signaling highlights the importance of controlling mechanical parameters in regenerative and soft robotic biomaterial systems designed to direct stem cell fate through physical cues.

### 3.4. Correlation Between Image-Derived Cellular Alignment and Finite Element Strain Fields

To quantitatively relate the observed cellular alignment to the mechanical environment of the stretch chamber, a finite element model (FEM) of the PDMS substrate was constructed. The model reproduced the geometry and boundary conditions of the custom-built stretching device, including the cyclic uniaxial displacement applied at the chamber ends. PDMS was modeled as an incompressible, hyperelastic material using a Mooney–Rivlin constitutive formulation with coefficients $C_{10}=0.12$ MPa and $C_{01}=0.03$ MPa, consistent with experimentally measured mechanical properties of Sylgard 184 at a 10:1 base-to-curing-agent ratio. The governing strain–energy function is given by

(3) $$W=C_{10}\left(I_{1}-3\right)+C_{01}\left(I_{2}-3\right),$$

where $I_{1}$ and $I_{2}$ are the first and second invariants of the right Cauchy–Green deformation tensor. From this constitutive relation, the local Cauchy stress and principal strain fields were extracted for each loading condition.

Simulations revealed a nearly uniform uniaxial strain field in the central region of the PDMS membrane, with strain magnitudes of approximately 10% and 1% corresponding to the two experimental stretching amplitudes. However, localized strain gradients were observed near the clamping boundaries, resulting in slightly elevated shear and transverse strain components in those regions. These non-uniformities provided a realistic representation of the mechanical microenvironment experienced by adherent cells, which typically span multiple finite elements across the substrate surface.

To directly compare the mechanical predictions with cellular behavior, the experimentally determined coherency index (*CI*) values from Table 2 were spatially correlated with the principal strain directions computed from the FEM results. In regions where the FEM predicted high uniaxial strain magnitude and low transverse shear, MSCs exhibited the strongest actin alignment (*CI* ≈ 0.85), consistent with alignment along the direction of maximum principal strain. Conversely, areas with lower strain amplitude or higher strain heterogeneity exhibited reduced alignment (*CI* ≈ 0.6), reflecting the dampened cytoskeletal organization under less coherent mechanical fields. This correspondence between numerical strain orientation and actin filament alignment supports the validity of the computational model in replicating the local mechanostimulus that drives cellular remodeling.

Furthermore, the model provided insight into the effective strain transmitted to the cell layer. Considering the finite thickness of the PDMS substrate (approximately 1 mm), the simulated surface strain closely matched the nominal actuator input, confirming that the cells experienced nearly full-strain transmission without significant damping or interfacial slip. This mechanical fidelity explains the high reproducibility of cytoskeletal alignment observed across samples and substantiates the assumption that the measured CI values are a direct functional readout of the applied substrate strain field.

Collectively, the FEM results complement the fluorescence-based image quantification by providing a mechanistic continuum-level context for cellular alignment behavior. The agreement between simulated strain orientation and experimentally derived alignment indices confirms that the cytoskeletal reorganization of MSCs serves as a faithful proxy for local substrate deformation. This integrated experimental–computational framework enables predictive mapping between mechanical loading regimes and cellular mechanoadaptation, establishing a foundation for designing soft robotic and biomaterial platforms with tunable mechanical microenvironments.

### 3.5. Mechanically Induced Osteogenic Differentiation of MSCs

**Figure 4:**
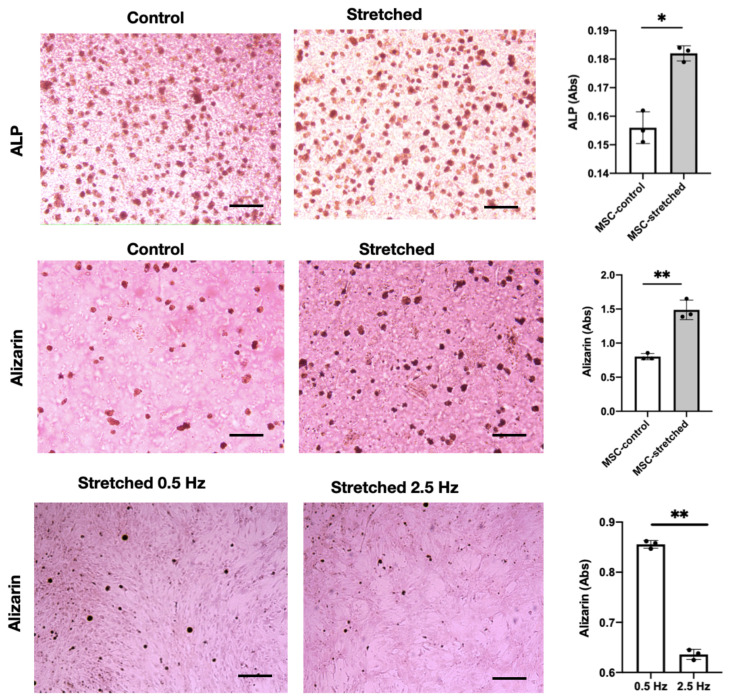
Alkaline phosphatase (ALP) and Alizarin Red S staining of MSCs encapsulated in Fmoc-FF peptide hydrogels under static and stretched conditions at frequencies of 0.5 Hz and 2.5 Hz. Stretched samples show higher ALP activity (*p* < 0.05) and increased calcium deposition (*p* < 0.01), indicating enhanced osteogenic differentiation under cyclic mechanical loading. Scale, 200 μm. *, p<0.05; **, p<0.01.

To evaluate whether cyclic mechanical stretching enhances osteogenic differentiation in mesenchymal stem cells (MSCs), both alkaline phosphatase (ALP) and Alizarin Red S (ARS) staining assays were performed after 14 days of culture under the optimized stretch condition (0.5 Hz, 10% strain). Figure 4 presents the staining results along with the corresponding quantitative assessments. In the ALP-stained images (top row), the stretched samples exhibited a clear rise in both the number of ALP-positive cells and staining intensity relative to the non-stretched controls.

Quantitative spectrophotometric measurements confirmed this observation, with the ALP absorbance value increasing from approximately 0.15 Abs in the control to 0.18 Abs in the stretched condition (p<0.05). An early rise in ALP activity is characteristic of cells progressing toward an osteoblast phenotype and corresponds with increased expression of early osteogenic indicators, such as *RUNX2* and *COL1A1*, as a result of sustained mechanotransductive signaling.

Subsequent Alizarin Red S staining (middle row) demonstrated a similar trend, with substantially higher calcium deposition in the stretched MSCs relative to the control. Mineralized nodules appeared more densely distributed and intensely stained, indicating greater matrix mineralization under cyclic loading. Quantitative analysis of the extracted ARS dye revealed that the absorbance increased from approximately 1.0 Abs in the control to nearly 1.8 Abs in the stretched samples (p<0.01). This twofold enhancement in calcium accumulation confirms that mechanical stimulation not only promotes early osteogenic signaling but also translates into functional extracellular matrix mineralization at later stages of differentiation. Comparison of MSCs stretched at 0.5 Hz and 2.5 Hz demonstrated that the 0.5 Hz condition resulted in higher calcium deposition, suggesting that lower frequencies favor osteogenic differentiation.

The combined results from ALP and ARS assays indicate that cyclic stretching at 0.5 Hz and 10% strain effectively accelerates the osteogenic commitment of MSCs. This outcome can be mechanistically attributed to the sustained activation of RhoA/ROCK and YAP/TAZ pathways described earlier, which facilitate cytoskeletal tension, nuclear deformation, and transcriptional activation of osteogenic genes. Moreover, the finite element modeling results showed uniform surface strain in the central region of the PDMS chamber, implying that cells experienced consistent mechanical stimuli—an essential factor for homogeneous differentiation across the culture area.

These findings collectively demonstrate that the mechanical microenvironment plays a decisive role in directing MSC lineage specification. The integration of quantitative fluorescence imaging, finite element modeling, and biochemical assays provides a coherent multiscale framework linking mechanical strain to cytoskeletal remodeling and ultimately to osteogenic functional outcomes. This experimental–computational approach underscores the potential of mechanically active substrates to guide stem cell differentiation for regenerative and soft robotic tissue engineering applications.

### 3.6. Upregulation of Osteogenic Gene Expression Under Cyclic Mechanical Stretch

**Figure 5:**
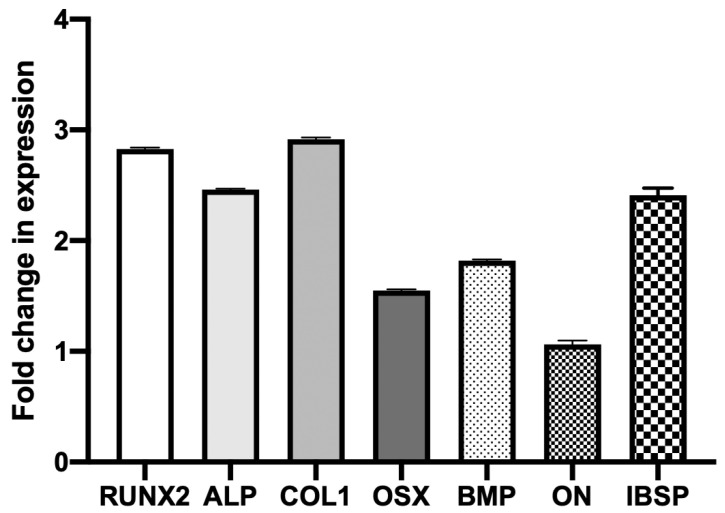
Fold-change analysis of osteogenic gene expression in MSCs subjected to cyclic mechanical stretch (0.5 Hz, 10% strain) compared to unstimulated controls. Early differentiation markers (*RUNX2*, *ALP*, *COL1*, and *OSX*) and late-stage mineralization markers (*BMP*, *ON*, and *IBSP*) were all upregulated, confirming enhanced osteogenic commitment under mechanical loading. Data represent mean ± SD (n=3).

To further elucidate the molecular mechanisms underlying osteogenic differentiation, quantitative real-time PCR (qRT–PCR) analysis was conducted on a panel of genes representing early, middle, and late stages of osteoblastic lineage commitment (Figure 5). The genes analyzed included early transcriptional regulators *RUNX2* and osterix (*OSX*), matrix-associated markers such as collagen type I (*COL1A1*) and alkaline phosphatase (*ALP*), and late-stage mineralization markers including bone morphogenetic protein (*BMP*), osteonectin (*ON*), and integrin-binding sialoprotein (*IBSP*). Gene expression values were normalized to the housekeeping gene *GAPDH*, and fold changes were determined using the 2^−ΔΔCt^ approach.

Mesenchymal stem cells (MSCs) encapsulated in 3D peptide hydrogels were subjected to cyclic mechanical stretching (0.5 Hz, 10% strain) and compared to static hydrogel controls. The results revealed significant upregulation of osteogenic markers across all stages of differentiation, with most genes demonstrating a 2–3-fold increase relative to static controls.

Among the early osteogenic transcription factors, *RUNX2* expression increased by approximately 2.8-fold, confirming its central role as a mechanosensitive regulator initiating osteogenic commitment. Similarly, *ALP* and *COL1A1* exhibited 2.5- to 3.0-fold increases, consistent with enhanced enzymatic activity and extracellular matrix synthesis observed in complementary biochemical assays. *OSX*, a downstream target of *RUNX2*, showed a 2.0-fold increase, reinforcing activation of early differentiation cascades through mechanotransductive signaling.

Late-stage markers associated with extracellular matrix mineralization also exhibited significant upregulation. Expression of *BMP* and *ON* increased by approximately 1.8-fold and 1.2-fold, respectively, while *IBSP* demonstrated the most pronounced response with nearly a 3.2-fold elevation in expression. The increased expression of these late-stage markers suggests that mechanical stretching not only triggers early osteogenic signaling but also sustains matrix maturation and mineral deposition over time.

The coordinated transcriptional activation of *RUNX2*, *COL1A1*, and *IBSP* supports the hypothesis that cyclic mechanical loading acts through mechanoresponsive pathways that couple cytoskeletal tension to gene regulation. As discussed previously, activation of RhoA/ROCK and YAP/TAZ under mechanical strain enhances actin stress fiber formation and nuclear deformation, facilitating nuclear localization of YAP/TAZ and subsequent transcription of osteogenic genes. Finite element modeling confirmed that the applied strain field was spatially uniform in the central region of the PDMS chamber, indicating that the observed gene expression patterns reflect a consistent mechanobiological stimulus across the cell population.

Previous finding has shown that cyclic mechanical loading promotes osteogenic differentiation of MSCs. For example, Sun [21] applied 8% strain at 1 Hz in a 2D system and observed increased matrix formation and early osteogenic marker expression, while Xiao et al. [24] demonstrated that intermittent stretching at 10% strain activated the p38MAPK–osterix pathway and enhanced osteogenesis in bone-marrow MSCs. Fang et al. (2019) used lower strain levels (3–5% at 0.5 Hz) in 2D culture and reported modest increases in proliferation and cytoskeletal organization, but significantly weaker osteogenic effects than those observed under higher-strain conditions [22]. Compared with these studies, which were focused on 2D monolayers in natural ECM, our 3D peptide hydrogel system subjected to 10% strain at 0.5 Hz elicited more pronounced cytoskeletal alignment and upregulation of osteogenic genes, likely due to the combination of higher strain magnitude and the physiologically relevant 3D matrix. This highlights the importance of both mechanical loading parameters and the 3D microenvironment in directing MSC osteogenic differentiation. Overall, these findings demonstrate that dynamic mechanical stimulation in 3D peptide hydrogels effectively promotes osteogenic lineage commitment of MSCs at both structural and molecular levels. The integration of qRT–PCR data with fluorescence imaging, finite element modeling, and biochemical assays provides a multiscale understanding of how external mechanical cues are transduced into gene-level responses.

## 4. Conclusions

This study demonstrates that dynamic mechanical stimulation within three-dimensional (3D) peptide hydrogels provides a powerful microenvironment for directing mesenchymal stem cell (MSC) fate. A custom-designed PDMS-based stretching device integrated with finite element modeling established a reproducible platform capable of delivering uniform cyclic strain to cell-laden hydrogels. Low-frequency, high-strain loading (0.5 Hz, 10%) induced significant cytoskeletal alignment (coherency index ≈ 0.85), enhanced actin polymerization, and increased cell proliferation compared to higher-frequency, low-strain conditions (2.5 Hz, 1%). Biochemical and gene expression analyses revealed a coordinated upregulation of early, mid, and late osteogenic markers—*RUNX2*, *ALP*, *COL1A1*, *OSX*, *BMP*, *ON*, and *IBSP*—confirming strain-dependent osteogenic differentiation. The findings indicate that mechanical cues in 3D peptide matrices activate RhoA/ROCK and YAP/TAZ pathways, coupling cytoskeletal tension to transcriptional control of osteogenesis. The integrated experimental and computational framework provides a multiscale understanding of mechanotransduction and underscores the potential of dynamically tunable peptide hydrogels for regenerative medicine and bone tissue engineering applications. These findings not only advance our understanding of MSC mechanotransduction in 3D matrices but also provide a foundation for translating mechanically optimized peptide hydrogels into preclinical bone-repair models and engineered tissue constructs. 

## Figures and Tables

**Table 1:** Primer sequences and qPCR performance parameters.

Gene	Primer Type	Sequence (5’–3’)	Amplicon Size (bp)	Efficiency (%)	Melt Curve Tm (°C)
*COL1A1*	Forward	GAT GGC TGC ACG AGT CAC AC	124	97.8	83.6
	Reverse	AGC CGA ATT CCT GGT CTG GG			
*BMP2*	Forward	CGC AGC TTC CAC CAT GAA GAA TC	136	99.5	82.9
	Reverse	GCC ACC ATG GTC GAC CTT TAG GA			
*ON (SPARC)*	Forward	GAG TGG TTT CCT GTT GCC TGT C	118	94.6	84.2
	Reverse	CAT GGT GCT GGG AAC CCT CAG			
*RUNX2*	Forward	ACC ACA GAA CCA CAA GTG CG	142	99.4	81.8
	Reverse	TGC TTG CAG CCT TAA ATG ACT CT			
*ALP*	Forward	GTG CTC TGC GCA GGA TTG GA	109	99.8	85.0
	Reverse	CAT GGT GCT GGA CCC CAA GAC CT			
*OSX (SP7)*	Forward	ACC GGA GCC TGA GTG GAA C	127	96.5	83.1
	Reverse	GCC ATA GTG AAC TTC CTC CTC A			
*IBSP*	Forward	AGG ACT GCC AGA GGA AGC AAT	115	92.8	84.7
	Reverse	CGT GGC GTC CTC TCC ATA GC			

**Table 2:** Quantitative metrics of cytoskeletal alignment, actin intensity, and cell density for MSCs under cyclic stretch. Values are expressed relative to unstimulated control (mean ± SD, n=3).

Condition	Coherency Index (*CI*)	Normalized F-actin Intensity ($I_{\mathrm{norm}}$)	Relative Cell Density
MSC Control	0.25±0.05	1.00±0.08	1.00±0.07
0.5 Hz, 10% strain	0.85±0.04	1.50±0.10	1.50±0.09
2.5 Hz, 1% strain	0.60±0.06	1.20±0.07	1.30±0.08

## Data Availability

The original contributions presented in the study are included in the article, further inquiries can be directed to the corresponding author.
